# Trends in diagnosis of painful neck and back conditions, 2002 to 2011

**DOI:** 10.1097/MD.0000000000006691

**Published:** 2017-05-19

**Authors:** Patricia L. Sinnott, Sharon K. Dally, Jodie Trafton, Joseph L. Goulet, Todd H. Wagner

**Affiliations:** a Health Economics Resource Center (HERC); b Center for Innovation to Implementation and Program Evaluation and Resource Center; c The Pain Research, Informatics, Multimorbidities and Education Center, VA Connecticut Healthcare System, West Haven, Connecticut; d Health Economics Resource Center (HERC) and Center for Innovation to Implementation, VA Palo Alto Healthcare System, Menlo Park, California.

**Keywords:** back pain, incidence, neck pain, prevalence

## Abstract

Neck and back pain are pervasive problems. Some have suggested that rising incidence may be associated with the evidence of rising prevalence.

To describe the trends in diagnosis of painful neck and back conditions in a large national healthcare system.

A retrospective observational cohort study to describe the incidence and prevalence of diagnosis of neck and back pain in a national cohort.

Patients were identified by International Classification of Diseases, 9^th^ Revision (ICD-9) codes in Department of Veterans Affairs (VA) national utilization datasets in calendar years 2002 to 2011.

Descriptive statistics were used to analyze the data. Prevalent cases were compared with all veterans who sought health care in each year. Incident cases were identified following a 2 years clean period in which the patient was enrolled and received care, but not services for any back or neck pain conditions.

From 2004 to 2011, 3% to 4% of the population was diagnosed with incident back pain problems, the rate increasing on average, 1.75% per year. During the same period, 12.3% to 16.2% of the population was diagnosed with a prevalent back pain problem, the rate increasing on average 4.09% per year.

In a national population, the prevalence rate for diagnosis of neck and back pain grew 1.8 to 2.3 times faster than the incidence rate. This suggests that the average duration of episodes of care is increasing. Additional research is needed to understand the influences on the differential rate of change and to develop efficient and effective care systems.

## 1 Introduction

Neck and back pain problems are pervasive and associated with chronic pain, disability, and high healthcare utilization.^[1,2]^ Among adults, 60% to 80% will experience back pain and 20% to 70% will experience neck pain that interferes with their daily activities during their lifetimes.^[3–5]^ At any given time, 15% to 20% of adults will report having back pain and 10% to 20% will report neck pain symptoms.^[3,6]^ The vast majority of back and neck pain complaints are characterized in the literature as non-specific and self-limiting, but with a high frequency of recurrence.^[6–9]^ Importantly, in low back pain, the duration of the first episode has been positively associated with the probability of recurrence, with each recurrence marked by increasing severity and disability.^[7,10]^ This suggests that what happens during the first or incident episode may have a significant influence on the occurrence and severity of subsequent episodes and the development of long-term disability. Additionally, back and neck pain consistently rank in the top five disabling disorders in the United States.^[11]^

Much has been written about the rising prevalence and costs of these conditions,^[1,2,12,13]^ which have been variously attributed to variation in reporting methods, changes in treatment patterns and the use of medical technologies, changes in patterns of care-seeking and combinations of the above.^[2,7,12,14–21]^ However, there is little reported about changes in care-seeking for incident episodes of these conditions primarily because the identification of a first event is complex for conditions so prevalent and recurrent^[22]^ and consistent historical data have not been available for such analyses.^[23–25]^ Research suggests that the prevalence of these conditions is increasing.^[12]^ Better understanding of changes in the diagnosis of incident episodes of care for back and neck problems, and the relationships between these first events and the rising prevalence will provide information critical to developing strategies to improve care for these patients.

Annually, VA provides health care to over 5 million veterans through the Veterans Health Administration (VHA) and its national system of providers (clinicians and facilities). It is the largest national healthcare system in the United States. Eligibility for VHA enrollment is determined by Congress, and is based on military service experience, medical, mental health, and physical consequences of such service and financial status. A large proportion of enrolled veterans do not qualify for other public or private health insurance. VHA administrative and electronic health record data have been collected for over 10 years and provide a consistent historical data resource for examination of the trends in diagnosis of painful back and neck conditions.

Previous research on VA patients has reported a population prevalence of low back pain ranging from 11%^[33]^ to as high as 52% (by self-report)^[26,27]^ and that almost 25% of patients who visit VA general medicine clinics suffer from chronic axial or spinal pain.^[28]^ Recent studies cataloging 42 chronic conditions in 2008 identified chronic low back pain as the 8th most prevalent condition in VA (8.1%) and growing.^[17,29]^

## 2 Methods

### 2.1 Design

This retrospective observational study sought to describe the trends in diagnosis of painful back and neck conditions in VA in calendar years 2002 to 2011. Using standard methods for identifying musculoskeletal spine pain from retrospective administrative data,^[30–32]^ we searched VA inpatient (patient treatment files) and VA outpatient (NPCD outpatient event and DSS outpatient national data extracts), and non-VA (fee-basis) utilization files for fiscal years 2002 to 2012 to identify patients who were diagnosed in 2002 to 2011. Patients were included in the analysis if they had an administrative record reflecting evaluation or treatment by any healthcare provider with an ICD-9 diagnostic code consistent with back or neck pain problems in any spinal segment.^[31,32]^ Neck pain was identified by ICD-9 diagnostic codes referring to the cervical spine. Back pain was identified using ICD-9 diagnostic codes specific to the thoracic, lumbar, sacral, or coccygeal spine. Codes that referred to the spine, but without reference to the anatomical segment were defined as “non-specific” spine pain problems. Because all cases were identified by ICD code, all cases were defined as either neck, back, or non-specific. We linked utilization data with the VA vital status file to define the patient's sex and age at the time of the incident encounter. Homelessness was defined, if at any time during the 1-year period prior to and including an included encounter the patient received treatment in a clinic for homeless veterans or was assigned a diagnosis code of V60.0. To identify the physical and mental health comorbidities of our populations, we accessed the HERC chronic conditions dataset, which identifies all patients with at least one encounter for a given condition in a fiscal year.^[29,33]^ Because the chronic conditions dataset are based on fiscal years, while our population was defined using calendar years, we linked the calendar year population to the chronic conditions in the corresponding fiscal year.

The incident encounter was defined as the first encounter with any healthcare provider in which one of the back or neck pain codes appeared in the data following a 2-year “clean period.” During the “clean period” the veteran was enrolled in VA healthcare (as indicated by the enrollment date contained in the VA Assistant Deputy Undersecretary for Health [ADUSH] enrollment files), used VA healthcare services, and had not had an encounter with any healthcare provider that included a diagnosis for a spine, back, or neck pain problem. An individual patient might have produced more than 1 incident encounter if the episodes of care were separated by 2 years or more. The denominator for the analyses includes all veterans who sought any care in a specified year. Non-veteran data (e.g., employees), which are sometimes included in the VA administrative records, were excluded from the analyses. Incidence is calculated as a count of each incident events in each fiscal year. Prevalence is calculated as the count of prevalent cases in each fiscal year.

We report patient demographics for all veterans who sought care, and for veterans who sought care for any back or neck pain problems, from 2002 to 2011. Because of the 2-year clean period, we report demographics for incident cases from 2004 to 2011. We report chronic condition comorbidities for all veterans and for veterans with prevalent and incident cases of neck or back pain in 2004 and 2011 and report the change over time in the distribution of these conditions. Chronic conditions are reported as cases, so that an individual patient may have more than 1 comorbidity. We report trends in care-seeking between 2004 and 2011 for prevalent and incident cases of neck, back, and non-specific spine pain problems by spinal segment in percent change over time, and in average annual change. Percent change over time is calculated using rate per thousand veterans. Because we are identifying the population of veterans with neck and back pain who used the VA, rather than analyzing a sample of cases, we omit reporting confidence intervals to simplify the presentation. Analyses were performed using SAS version 9.2 (SAS Institute Inc. Cary, NC). The study was approved by the Stanford Institutional Review Board.

## 3 Results

### 3.1 Sample characteristics

The patients who sought care for spine pain problems, consistent with the VA general population, were almost entirely men, but were also 2 to 4 years younger and more likely to be homeless than the general population. Also consistent with the VA general population, the mean age of the population with spine pain declined over the 9 years of the study and the women proportion of the population increased (Table 1). The percent of the population that sought care for any spine-related problem (e.g., back and neck and unspecified combined) grew from 15% of the total population in 2002 to 24% of the population in 2011, a 55% increase (Table 1).

**Table 1:**
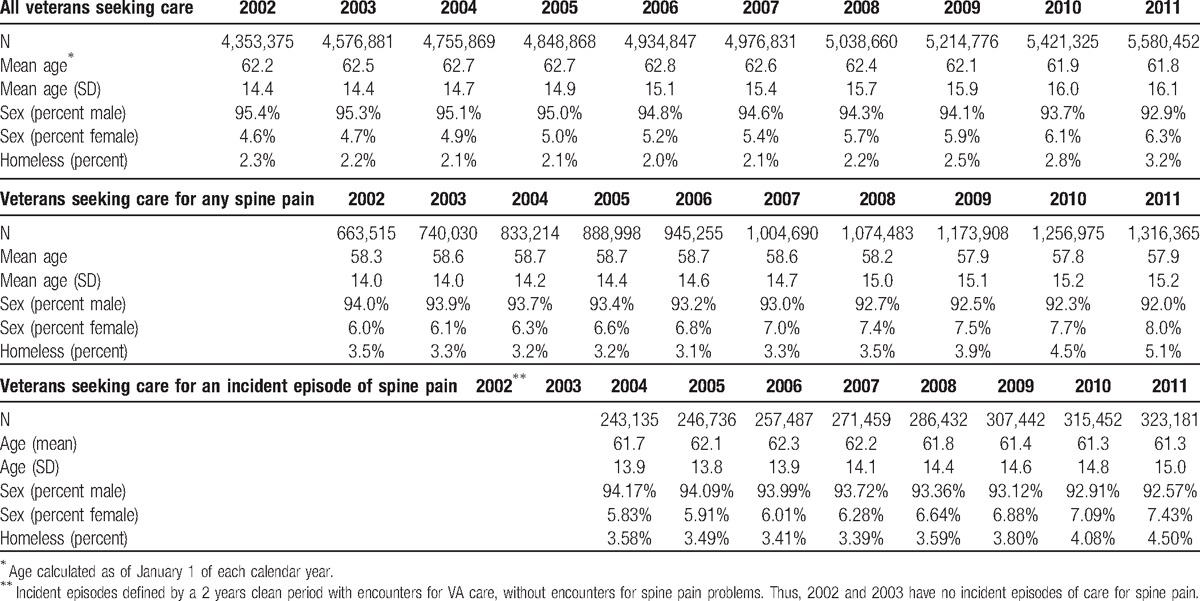
Demographics of all veterans seeking care, veterans seeking care for spine pain and veterans seeking care for an incident episode of spine pain, 2002 to 2011.

### 3.2 Comorbidities

Patients with encounters for spine pain problems had a similar prevalence of chronic hypertension, cardiovascular diseases, and diabetes to the reference population, but were more likely to have arthritis, headaches, and osteoporosis, and tobacco, alcohol, and any drug dependence/abuse disorders. The rate of change in the prevalence of these chronic conditions from 2004 to 2011 was greater in the general population for all chronic conditions except for hypertension, arthritis, congestive heart failure, renal failure, osteoporosis, cocaine and opium dependence, any drug dependence, manic depression, and schizophrenia which grew faster in the spine pain population (Table 2). Importantly, in 2011, the population with new spine pain encounters was 30% more likely to have depression or posttraumatic stress disorder (PTSD) than the general population and those with prevalent cases were approximately 60% more likely to have either of these conditions (Table 2).

**Table 2:**
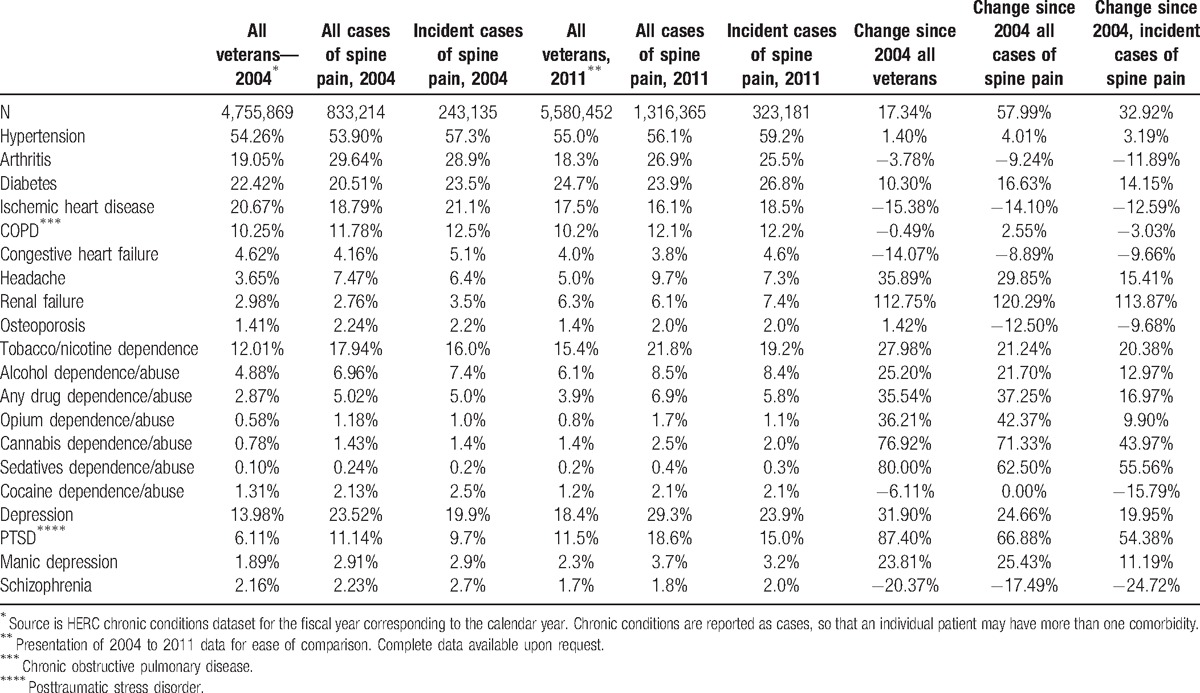
Comorbidities^∗^ of veterans seeking care for spine pain and comparison 2004 to 2011, in percents.

### 3.3 Diagnosis of incident and prevalent episodes of spine pain

Between 2004 and 2011, 3.8% to 4.3% of the veteran population was diagnosed with an incident episode of back pain, 1% for an incident episode of neck pain, and less than 1% of the population was diagnosed with a new episode of non-specific pain or pain in multiple segments (Table 3). The diagnosis of incident cases of back pain grew on average 1.75% per year while the diagnosis of incident cases of neck pain and incident cases of problems in multiple spinal segments grew 2.08% and 1.67%, respectively. Only the diagnosis of incident cases of non-specific spine pain declined, on average −1.4% per year.

**Table 3:**
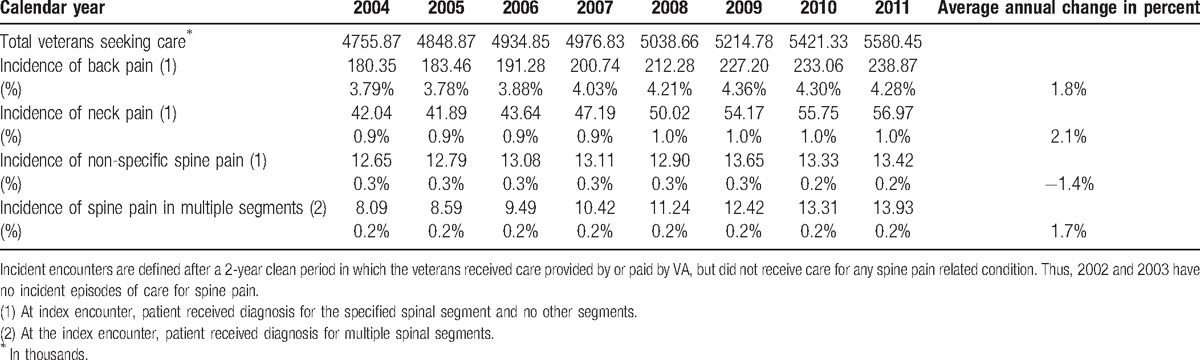
Care-seeking (in thousands) for incident episodes of spine pain, 2004 to 2011.

In contrast, from 2004 to 2011, the number of patients diagnosed with prevalent cases of any back pain (incident and non-incident) grew from 12.3% to 16.2% of the population, with neck pain from 1.9% to 2.5% of the population, and with pain in multiple spinal segments from 2.6% to 4.2%. Diagnosis of patients with non-specific spine pain was essentially unchanged (0.7%). These increases translate into a more than 50% increase in the absolute number of patients diagnosed with back pain (55%), or neck pain (53%), an 11% increase in the population diagnosed with non-specific spine pain problems, and an 87% increase in the population seeking care for pain in multiple spinal segments. These increases also reflect average annual increases in diagnosis of prevalent back and neck pain problems of 4.1% per year for back pain, 3.8% for neck pain, and 6.9% for pain in multiple segments (Table 4).

**Table 4:**
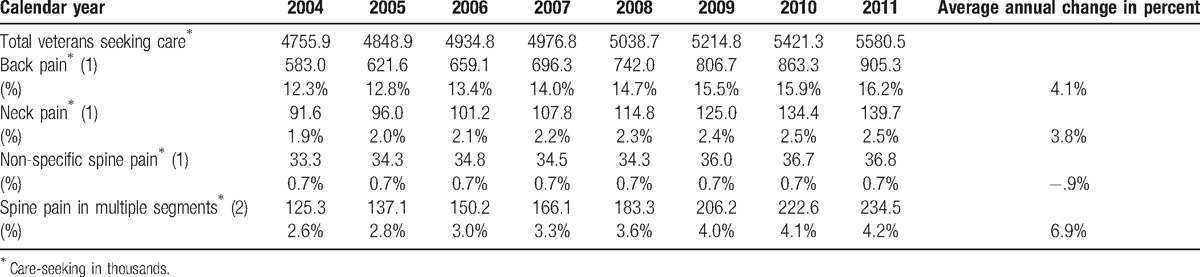
Care-seeking (in thousands)^∗^ for prevalent cases of neck and back pain in VA, calendar years 2002 to 2011.

## 4 Discussion

**Figure 1:**
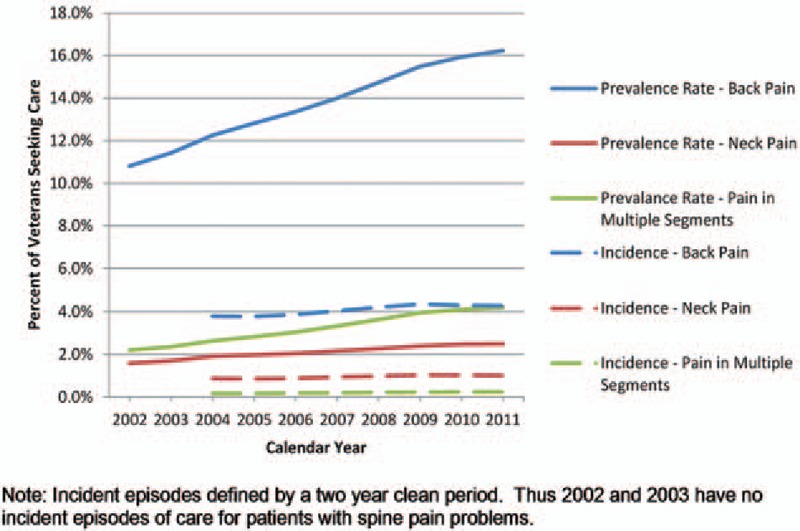
Trends in diagnosis of patients with incident and prevalent spine pain problems.

Back and neck pain problems are frequently reported in the literature as highly prevalent, costly, and poorly understood. Recent studies have reported increasing prevalence^[12,14]^ hypothesizing that this might be associated with a variety of factors, including increasing obesity,^[34]^ depression,^[35]^ and changes in symptom reporting. Less is known about the secular trends in incidence, and whether these trends mirror changes in prevalence. In this study, we show that between 2004 and 2011, approximately 4% of the veteran user population were diagnosed with a new back pain complaint, while an additional 8% to 12% of the population were diagnosed with ongoing or recurrent problems. The diagnosis of incident back or neck pain events grew at an annual average of 1.8% to 2.1% per year, while the diagnosis of prevalent cases of back and neck pain grew nearly twice as fast. The increase in the diagnosis of both incident and prevalent cases (see Fig. 1) is of particular concern because of the increasing economic burden these conditions represent. Chronic low back pain consistently ranks among the top most prevalent and expensive conditions managed by health care systems, and cost per episode is growing.^[14,29,33,36]^ The demands to health care systems for this care are multifaceted^[37–40]^ and there is a very strong association between mental health and substance use disorders and chronic pain.^[41–43]^ In particular, for VA, these risks are important because of the high prevalence of painful back and neck conditions in veterans returning from Operation Enduring Freedom/Operation Iraqi Freedom deployments,^[44,45]^ and the greater prevalence of painful musculoskeletal conditions in women compared with men.^[37,46,47]^

Of particular importance are the differential trends in the rate of change between the diagnosis of incident and prevalent cases of back and neck pain. The data show faster growth in prevalent cases compared with incidence cases; in 2004 the difference between the percent of the population with incident and prevalent cases of back pain was 8.5% while the difference in 2011 was 11.9%—a 40% increase. Similarly, the difference in the proportion of the population diagnosed with incident and prevalent neck pain increased 50% from 2004 to 2011. This increasing divergence in diagnosis rates suggests that the average duration of care for these problems is increasing.^[48,49]^ This increasing duration of care might be associated with changes in risk factors (e.g., depression or injury severity) or with changes in symptom reporting or treatment patterns. It may also be that the increasing duration is related to drug seeking behavior or a desire to claim disability. Examining these explanations is beyond the scope of this study, but more research is clearly warranted given the economic implications of prolonged utilization and progressive disability. However, these results do suggest that the increasing prevalence is not merely a function of rising incidence. Understanding and altering the factors that contribute to the conversion of an incident episode to a chronic condition will have a significant impact on this unsustainable upward trend in prevalence.

This is the first population-based study to compare trends in the diagnosis of both incident and on-going cases of neck and back pain in a single national population. While limited to veterans eligible for VA healthcare, and limited to the care they sought within the VA healthcare system, there are particular strengths to these analyses. The VA healthcare system is the largest healthcare system in the United States with a uniform administrative structure and little or no perverse financial incentives to influence provider behavior. Additionally, this format for evaluating both the incidence and prevalence of care-seeking for painful back and neck conditions provides a framework for the population health management critical to managing and improving the care for a large population of patients. Importantly, these results and our methodology are likely to be of interest to accountable care organizations with similar financial and administrative structures and population health management goals.

The results suggest that increasing duration of care and not changes in the diagnosis of incident back and neck pain conditions are driving the increasing prevalence of these problems. Next steps in this area of research include examination of the influence of risk factors, guideline adherence, and treatment on episode duration and patient recovery and comparison of these findings in the veteran population to other national cohorts.

As with other retrospective cohort studies there are potential limitations to this study. First, we were unable to include data from non-VA sources for these patients hence we may have characterized follow-up encounters in VA as incident events. However, this would not lead to under or over counting encounters for the VA health care system to manage and is consistent with methods that might be used in other health care systems for identifying incidence of these problems. Additionally, we have required a 2-year clean period for the incident cases, which necessitates exclusion of incident cases from the 2002 and 2003 reporting. However, the very slow growth in incident cases in the following years, suggests that the ratio between incident and prevalent cases in these years (and the demand on the health care system) is similar to the following years. What might be missing are data from patients who chose or were able to seek care for these conditions entirely outside of VA. This would lead to an undercount of veterans with back or neck pain, but not an underestimation of the number of cases managed by the healthcare system. Finally, we have made assumptions about the presence of pain for these patients based on ICD-9 codes, which is consistent with the literature, but does not reflect examination of patient reported pain scores. We also do not know if the presence of these ICD-9 codes in the encounter data reflects the presenting condition for which the patients sought care at any one encounter. Research linking administrative data to clinical chart data was beyond the scope of this study, but will be necessary to confirm these findings.

## 5 Conclusion

In a national population, diagnosis of on-going or prevalent cases of neck and back pain is rising two times faster than diagnosis of new or incident cases, suggesting that the average duration of care for these patients is increasing. Additional research is needed to understand the influence of demographic factors, the adequacy of treatment, and guideline adherence on the outcomes for patients and to develop effective and efficient healthcare management systems.
